# The value of clinical audit to improve cataract quality

**Published:** 2014

**Authors:** Robert Lindfield

**Affiliations:** Clinical Lecturer: The Disability and Eye Health Group, London, UK Robert.Lindfield@Lshtm.ac.uk

**Figure:**
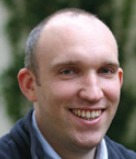
Robert Lindfield

The *Community Eye Health Journal* is publishing a series of articles about clinical quality. The main focus of these articles will be practical ways to monitor and improve clinical quality using examples from hospitals in a range of low- and middle-income countries. Do share your examples with us and we will publish as many as possible.

One of the easiest ways to start improving quality in a hospital is through clinical audit. Clinical audit is a quality improvement process that seeks to improve patient care and outcomes. It does this through a systematic process of review or evaluation against clearly defined criteria, followed by the implementation of change.

Clinical audit is something that doctors, nurses, optometrists and clinical officers can take part in; and they should be encouraged to do so.

## Audit cycle

**Figure 1.**
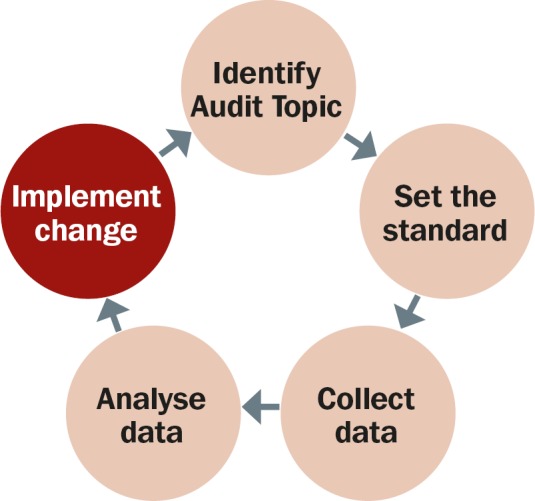
The audit cycle

There are five steps in an audit cycle (Figure 1). Once the last step is complete, the cycle begins again (called re-auditing).

### 1 Identify the audit topic

Is there an issue or problem in the hospital that you think is important? The topic of the audit might be a very simple one, for example whether the wards are clean (e.g., do patients think the wards are clean?) or whether staff have received specific training (e.g. what proportion of staff members have received training in post-exposure prophylaxis for needle stick injuries?). It can also be very complex, for example whether patients have good visual acuity (VA) after surgery (this is complex because VA after surgery depends on several factors).

### 2 Set the standard

Is there a standard for performance related to this topic? For example, do we want all patients to say that the wards are clean? Or 90%? What is realistic? Should 100% of staff have had training in needle stick injuries? Or is 50% adequate? Some topics have international standards. For example, the World Health Organization (WHO) recommends that 90% of cataract operations should have a visual outcome better than 6/18. You need to decide what standard you think is appropriate.

### 3 Collect data

This might be as simple as asking all staff members to tell you whether they have received training, or speaking to every patient who is discharged during one week to find out how many thought the wards were clean. Collecting data from patient records is a bit more complicated. It helps if you have access to electronic patient records, e.g. to work out the percentage of patients who had VA better than 6/18 du ring the course of one month.

### 4 Analyse data and draw conclusions

You need to compare what you've found with what you set as your ‘standard’, and decide what to do next. E.g., what if you thought that every member of staff should have received training and only 80% had? What if you wanted 90% of patients to report that the wards were clean and only 20% said they were? What if only 60% of patients achieved a visual acuity better than 6/18 after cataract surgery when the WHO recommends that 90% achieve this?

### 5 Implement change

This is the most difficult part of clinical audit but it is the most important. It is an opportunity to try something simple and find out whether it works. Sometimes the solution is obvious – if staff have not received training on needle stick injuries then they need to attend training. If patients feel that the wards are not clean then they need to be cleaned. Sometimes, however, the solution is not obvious – how do you improve the percentage of people achieving VA of 6/18 after cataract surgery?

The most important part of a clinical audit is to make sure that, if your initial changes are not successful, you go back and try something else. You can then check whether that has been successful through re-auditing. This means repeating your audit and finding out whether anything has improved.

As a hospital it is useful to have a standard set of audits that you carry out during the year. It takes time to complete an audit, so staff must be given time off from their usual duties. They will also need to share their findings with other clinical staff so there should to be meetings or workshops where the results of the audit can be shared. That said, the cost of an audit is usually very low and the results are, potentially, very valuable.
